# Topics of the Month

**Published:** 1895-02

**Authors:** 


### TOPICS OF THE MONTH.

The third annual report of the Sheppard Asylum, a hospital for mental diseases, located at Towson, Baltimore Co., Md., has had under treatment 117 patients during the past year, the daily average under care being sixty. During the year fifty-seven patients have been discharged, fourteen of whom have recovered and eighteen much improved or improved. Nine deaths have occurred at the hospital during the year, two of which were in a hopeless condition when admitted.

An interesting fact recorded in this report is, that during the year twenty-three patients have applied voluntarily for treatment and have been admitted to the hospital. Of these, eight were cases of alcoholic or narcotic addiction. The others were suffering in a greater or less degree with active mental disturbance, varying from incipient melancholia to chronic delusional insanity or paranoia.

The report is handsomely illustrated with views of the buildings and grounds. This institution is presided over by Dr. Edward N. Brush, who is physician-in-chief and superintendent, and who is to be congratulated upon the result of another year's work so interestingly recorded in this pamphlet.

———

In these columns we have before referred to the fact that an attempt is making in certain quarters to substitute the word celiotomy for that of laparatomy as a surgical term. We hold to the opinion, as heretofore expressed, that both being wrong it is better to maintain the expression, abdominal section, until a better term is found,—one more correct, at least, than either of the two referred to.

Under the lead of Dr. Robert P. Harris, of Philadelphia, the members of the American Gynecological Society, in large numbers, have adopted the substitute and appear to be seeking to incorporate it permanently in the literature of medicine. Many younger men also, not members of the society referred to, are easily following its lead. The argument showing its error, etymologically and surgically, has been frequently referred to in journalistic print, but nowhere have we seen it more effectively set forth than in a letter by Dr. R. Stansbury Sutton, of Pittsburg, published in the *New York Medical Journal*, December 29, 1894.

Dr. Sutton, himself a member of the American Gynecological Society, says:

"Laparotomy" as a surgical term is wrong, but it should be borne in mind *that technical terms acquire validity from long usage, often in spite of wrong etymology.* If, therefore, it can be shown that the term cœliotomy is also an incorrect term etymologically, we do not improve matters by rejecting for it the term laparotomy. My Greek is very rusty; it is thirty-two years since I was graduated at old Jefferson College, at Canonsburgh, Pa., but I will try with what Greek is left these two words, "laparotomy" and "cœliotomy," and the reader may decide for himself on the merits of the case.

*Lapara* in Greek is derived from an adjective which means *slack* or *loose*, and in the human body is applied to the *flank*, meaning the loose flesh between the ribs and the pelvic bones or the hip. In military parlance it is a technical synonym for our word *flank*.

From *lapara* and the verb *temno*, to cut, is derived the word laparotomy. Literally it means to cut the *flank*. *It has acquired validity from long usage*, but is etymologically incorrect.

*Cœlia* is the Latin, from the Greek word *koilia* derived the Greek *koilos*, which means *hollow*, and in the human body means a *hollow viscus*, and was applied to the abdomen, heart, uterus, intestines, thorax and stomach, etc.—any cavity of the body. Hippocrates applied it to the socket of the hip joint. The Greek adjective *koiliacus* was used to refer to anything contained in the abdomen or in any of its viscera—fæces, for instance. From this word *koilia* and the verb *temno* is derived the word cœliotomy. It means literally to cut open a *hollow viscus*, which the abdomen is not. It is no better than the term laparotomy, *and lacks the validity from long usage* which belongs to laparotomy. I believe the English term "abdominal section" is free from objection.

The importance of speaking and writing correct medical English and all derivative terms is sufficiently great to warrant this extended reference to a somewhat worn subject.

---

It is three years since the present health department took office, hence a comparison between the death rates under the present regimé and those of its predecessor will be in order.

In 1891 the death-rate was 23.46 per 1,000, which was the last year under the old charter; in 1892 the rate was 19.98 ; in 1893 it was 19.03, and in 1894 it was 17.76.

This reduction has taken place during a period in which the city has been steadily and largely increasing in population year by year, and endangered by surrounding and threatened epidemics. In spite, too, of the fact that typhoid fever prevailed as an epidemic during the months of February, March and April, 1894, in which period as many as a hundred cases were reported in a single day, that year shows the largest decrease in death rate. The cause of the typhoid epidemic alluded to was early recognized through the bacteriological researches of the department, and prompt measures taken through timely warning to the people, as well as in other ways, to control and limit the spread of this water-borne disease.

These comparisons are not made to cast any reflection upon the personnel of the old system, but to show how efficient the working of the health department has been under the enlarged powers granted in the new charter.

One of the ways in which the death rate has been reduced during the last year was through greater attention given our milk supply, compelling dealers in this commodity to refrain from keeping their milk in barns, near outhouses, and in like unwholesome places. The coolers are now kept in buildings at a distance from water closets and sewers, and are properly trapped and vented in order to prevent all possible contamination.

---

In St. Louis a plan is adopted for conveying patients from various portions of the city to the city hospital by means of an electric railway ambulance. According to *The Clinique* a street car with electric motor attachments has been equipped as an ambulance, and is running to all parts of the city in response to ambulance calls at an average speed of twelve miles an hour. We have already called attention to this improvement in these columns and have suggested its adoption in Buffalo.

With a completely equipped system of electric railways, reaching in all directions, and permeating to the most remote points, there seems to be no good reason why this humane plan should not be put in operation. It is not very long since a horse ambulance going at a high rate of speed down our principal business street cost a human life. If electric railway ambulances were adopted, this danger would be obviated, to say nothing of the greatly increased comfort that would accrue in the transit of the sick and injured.

We hope the health department will present this subject with such cogent force to the street railway management as to secure its prompt adoption.

---

Medical pronunciation is an important subject to the progressive and polished physician. It is not only pertinent to use correct medical terms, as indicated by our comments in another " topic " in this issue, but it is equally essential to pronounce them properly.

The *Western Reserve Medical Journal* has lately made forceful comment on the subject, as follows :

An unfortunate uncertainty obtains among the profession of this country as to the proper pronunciation of the large class of words whose termination is—*itis*. Many prefer the continental vocalizing of *i*. giving it the sound of English *ē*. The English profession and a very respectable portion of the American profession use the vowel *i* with its long English phonation—*ī*. There can be little doubt of the correctness of the usage of the latter group, so long as it is English we speak and not German or French. There is absolutely no authority for a continental vocalization of *i* in English speech, for if custom is pleaded in its favor it is easily shown that custom is widely at variance and that "—*ītis*" has more users than "*ētis*." Neither is there any uncertainty among the lexicons, whether medical or general, for with quite remarkable unanimity they give the English phonation. Expressing our sympathy for the adherents of the continental sound, we must needs leave them to their fate.

